# 2574. Serotype Distribution of *Streptococcus pneumoniae* and Pneumococcal Vaccine Coverage in Adults in Turkey between 2019 and 2022

**DOI:** 10.1093/ofid/ofad500.2191

**Published:** 2023-11-27

**Authors:** Gülşen Hasçelik, Münevver U Hasdemir Gökboğa, Gülşen Hazırolan, Yasemin Ay Altıntop, Lütfiye Öksüz, Murat Telli, Cüneyt Özakın, Sabire Ş Aydemir, Betil Özhak Baysan, Asuman Birinci, Akgün Yaman, Gül DURMAZ, Esra Gövde Özkaya, Zeynep Çizmeci, Mahmut Mete, Özgen A Özbek, Hakan Uslu, Candan Öztürk, Hüseyin Güdücüoğlu, Yücel Duman, Yasemin Zer, Eda Karadağ Öncel, Adem Karbuz, Mehmet Ceyhan

**Affiliations:** Hacettepe University Faculty of Medicine, Ankara, Ankara, Turkey; Marmara University Pendik Training and Research Hospital, Istanbul, Istanbul, Turkey; Hacettepe University Faculty of Medicine, Ankara, Ankara, Turkey; Kayseri City Training and Research Hospital, Kayseri, Kayseri, Turkey; Istanbul University, Istanbul Faculty of Medicine, Istanbul, Istanbul, Turkey; Aydın Adnan Menderes University, Faculty of Medicine, Aydın, Aydin, Turkey; Bursa Uludag University, Faculty of Medicine, Bursa, Bursa, Turkey; Ege University, Faculty of Medicine, Izmir, Izmir, Turkey; Akdeniz University, Faculty of Medicine, Antalya, Antalya, Turkey; Samsun Ondokuz Mayıs University, Faculty of Medicine, Samsun, Samsun, Turkey; Cukurova University, Faculty of Medicine, Adana, Adana, Turkey; Eskisehir Osmangazi University, Faculty of Medicine, Eskisehir, Eskisehir, Turkey; Karadeniz Technical University, Faculty of Medicine, Trabzon, Trabzon, Turkey; Istanbul Bakırköy Dr. Sadi Konuk Training and Research Hospital, Istanbul, Istanbul, Turkey; Dicle University Faculty of Medicine, Diyarbakır, Diyarbakir, Turkey; Dokuz Eylul University, Faculty of Medicine, İzmir, Izmir, Turkey; Atatürk University Faculty of Medicine, Erzurum, Erzurum, Turkey; Mersin University, Faculty of Medicine, Mersin, Mersin, Turkey; Trakya University Faculty of Medicine, Edirne, Edirne, Turkey; İnönü University, Faculty of Medicine, Turgut Özal Health Center, Malatya, Malatya, Turkey; Gaziantep University, Faculty of Medicine, Gaziantep, Gaziantep, Turkey; İzmir University of Health Sciences İzmir Tepecik Training and Research Hospital, İzmir, Izmir, Turkey; Istanbul Prof. Dr. Cemil Tascioglu City Hospital, İstanbul, Istanbul, Turkey; Hacettepe University Faculty of Medicine, Ankara, Ankara, Turkey

## Abstract

**Background:**

*Streptococcus pneumoniae* infections are challenging due to pneumococci having more than 100 serotypes. This ongoing study is aimed to evaluate serotype distribution of *S. pneumoniae* causing pneumococcal infections in adults ( >18 years) and to provide a perspective regarding serotype coverage of both current and future pneumococcal vaccines.

**Figure:**
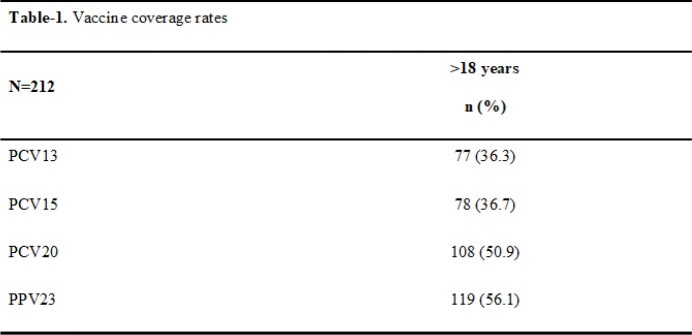

**Methods:**

Pneumococcal strains were collected from 22 centers between January 2019 and December 2022. Serogrouping was performed using latex particle agglutination, and serotyping was performed by the conventional Quellung reaction using commercial type-specific antisera (Statens Serum Institut, Copenhagen, Denmark).

**Results:**

During the study period, 212 pneumococcal strains (156 males [73.6%] and 56 females [26.4%]) were collected from adults over 18 years of age. Of the isolates, 114 (53.8%) were from patients < 65 years old and 98 (46.2%) were from patients ≥65 years old. The majority of isolates were obtained from blood (n=123, 58%) followed by bronchoalveolar lavage [BAL] (n=38, 17.9%) and cerebrospinal fluid (CSF) (n=25, 11.7%). The most frequently isolated serotypes were 3, 9N and 8. The vaccine coverage for PCV13, PCV15, PCV20, and PPV23 was 36.3%, 36.7%, 50.9%, and 56. 1%, respectively, in all isolates. The vaccine serotype coverage rates in the study group are shown in Table-1.

**Conclusion:**

It has been observed in time that while vaccine serotypes are decreasing, serotypes not covered by vaccines are increasing. Accordingly, surveillance is essential to determine vaccination policy.

**Disclosures:**

**Mehmet Ceyhan, n/a**, Pfizer Pharmaceuticals, Istanbul, Turkey: Advisor/Consultant|Pfizer Pharmaceuticals, Istanbul, Turkey: Grant/Research Support

